# Culturally sensitive mental health research: a scoping review

**DOI:** 10.1186/s12888-025-06575-z

**Published:** 2025-03-03

**Authors:** Phoebe Lyons, Auden Edwardes, Laura Bladon, Kathryn M. Abel

**Affiliations:** The University of Manchester, Manchester, UK

**Keywords:** Cultural Competence, Cultural Sensitivity, Mental Health Research, Mental Health, Health Inequality, Ethnic Minorities, Research Methodology

## Abstract

**Background:**

Given disparities in healthcare outcomes between ethnocultural groups, there is a need for research to be sensitive to cultural needs and differences to consequently address health inequalities. As part of a project aiming to co-produce a publicly acceptable core mental health dataset (CMHDS) for use in physical health research, evidence was sought as to how this could be made inclusive of a diverse population. The objective was to answer the following questions: What is best practice for culturally sensitive mental health research? How should mental health research be adapted to be culturally sensitive?

**Methodology:**

A comprehensive literature search was conducted using the following four electronic databases: PsychINFO, EMBASE, CINAHL and Medline as well as a capped Google search (using both the generic search engine and Google Scholar). Articles were screened at the title and abstract level and at full text by two reviewers. Key topics and conclusions from the included papers were tabulated and grouped in order to identify themes.

**Results:**

575 results were identified from the database searches after removing duplicates, which were narrowed down to 95 potential studies after the first round of screening by title and abstract. Full text article review in the second round of screening resulted in a total of 41 included articles for the final analysis. The 100 Google results were all assessed for eligibility with 2 being included. In total, 43 results were identified of relevance and included in the review. Overarching themes identified were as follows: the definition of ethnocultural groups, the impact of language, the influence of research team composition, appropriate research methodologies, and ethical responsibilities of researchers.

**Conclusions:**

The authors conclude that culturally sensitive research requires ongoing commitment to critical analysis, self-reflection, and collaboration from both researchers and institutions. Ethical considerations, including the historical context of racism and colonisation in mental health research, must be acknowledged to build trust and ensure that research outputs are equitable and applicable to diverse populations.

**Supplementary Information:**

The online version contains supplementary material available at 10.1186/s12888-025-06575-z.

## Introduction

In the UK, Black women and men have the highest rates of common mental disorders, with Asian women and Mixed race women also having higher rates of common mental disorders than White British women [1]. These outcomes are also seen in severe mental illness such as psychosis, which has the highest prevalence among second generation African-Caribbean people [2]. Black and ethnic minority groups are more likely to be offered medical treatments for mental illness as opposed to psychotherapy and to be treated with coercion in the UK– even when socioeconomic status is taken into account- with rates of psychiatric admission three or more times higher than average in black African, black Caribbean, and white and black Caribbean mixed groups [2]. It has been suggested that these findings are, at least in part, the result of institutional racism, with systems influenced by prejudice, ignorance and stereotyping [2]. More rigid clinical responses may be derived from reliance on a medical model, mistaking culturally different behaviour for illness [2]. Given this relationship between cultural misunderstanding or prejudice, diagnosis and treatment, it is important that researchers and clinicians work to build services and research practices which are sensitive to cultural needs and differences.

Those who historically have been most at risk of poor mental health are also those who were exposed to the greatest risk of death and financial insecurity throughout the COVID-19 pandemic [3]. These difficulties increase vulnerability to mental illness and suggest that, now more than ever, culturally sensitive mental health research is crucial to avoid deepening health inequalities [3].

The CMHDS project (NIHR 201104) aimed to co-produce a publicly acceptable core mental health dataset (CMHDS) for use in physical health research to deepen understanding about the links between mental and physical health. In order to draw out best practice and to highlight the ways in which our research could be underserving certain communities, we have undertaken an international scoping review on culturally sensitive mental health research. Insodoing, we sought to answer the following questions: What is best practice for culturally sensitive mental health research? How should mental health research be adapted to be culturally sensitive?

This work will ensure that the final CMHDS is relevant to all populations in the UK and is designed by, and for diverse populations. In this way, it addresses the National Institute for Health Research and the Clinical Research Network’s priority to deliver research that is inclusive and non-discriminatory [4].

## Methodology

### Search strategy

A comprehensive literature search was conducted using the following four electronic databases: PsychINFO, EMBASE, CINAHL and Medline. It was considered prudent also to include grey literature, recognising that this topic may be underrepresented in traditional academic manuscripts and, therefore, a search was also conducted using Google Scholar. The searches were conducted in January 2023. The search strategy comprised of keywords, synonyms, and controlled vocabulary (subject / MeSH headings) relating to the two related concepts of ‘cultural sensitivity’ and ‘mental health research’. An example of the search strategy (which was modified as necessary for specific databases) is available in the Supplementary Material. A Google search was performed asking ‘What is best practice for culturally sensitive mental health research?’ and the first 100 results screened for relevant content.

### Selection criteria

There was no limitation to language or year of publication. Studies were excluded if they referred to white minorities or to only ‘migrants’. Literature reviews and systematic reviews were excluded. The focus had to be on conducting mental health research, rather than clinical assessment and/or treatment. Other than excluding systematic reviews and literature reviews, there was no further exclusion by study design. Opinion pieces were included, recognising the value of experts by experience and with the aim of including voices which may not traditionally be heard in academia.

### Screening and study selection

Database search results were collated using the reference management software Rayyan. Two reviewers (PL, LB) independently checked the titles and abstracts for inclusion. Conflict was resolved by discussion between the two reviewers to reach a consensus. A third researcher (AE) checked their findings. helping to ensure the search was conducted rigorously.

## Results

**Fig. 1:**
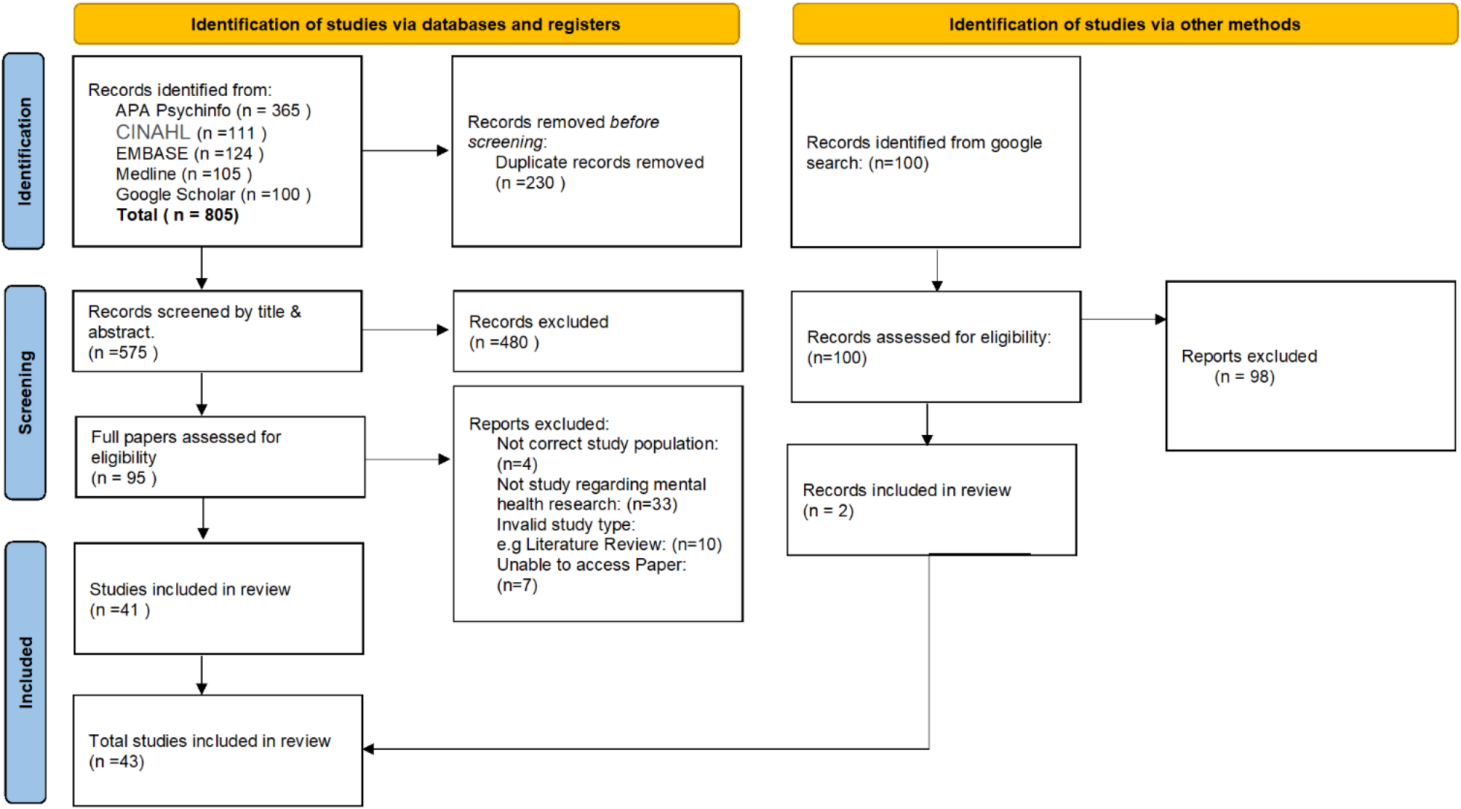
Preferred Reporting Items for Systematic Reviews and Meta-Analyses (PRISMA) flow diagram

We identified 575 results from the database searches after removing duplicates, which were narrowed down to 95 potential studies after the first round of screening by title and abstract. Full text article review in the second round of screening resulted in a total of 41 included articles for the final analysis. The 100 google results were all assessed for eligibility with 2 being included. In total, 43 results were identified of relevance and included for the narrative review. Figure 1. shows the study selection procedure in PRISMA format.

Of those 43 studies, authors were affiliated with institutions from twelve different countries which were as follows: Australia, Botswana, Canada, China, Ethiopia, Ghana, India, Iran, Nigeria, South Korea, UK and US. The results spanned a time frame from 1989 to 2022.

Due to the heterogeneity of the results, tools of comparison were not appropriate to use. The data was largely qualitative and hence results were reviewed using thematic analysis. The papers were tabulated with summaries of ideas and conclusions for each one; these were then grouped based on similar patterns and topics, and subsequently themes were identified and explored.

## Identified themes

### Defining ethnocultural groups

Ethnocultural groups are groupings of people who share a common ethnicity and culture, often including religion and linguistics. Frequently in research ‘ethnic glosses’, broad terms based on ethnic group (e.g. Hispanic) - are used to categorise, which may reflect a diverse and heterogenous population within them who do not necessarily have a shared culture [5]. Even controversial and stigmatising terms such as ‘non-white’ continue to appear in published literature [6]. Hall points out that, within ethnic minority groups, those who identify most strongly with the ‘mainstream’ about language and acculturation are most likely to participate in research [7]. When aiming to recruit from a particular ethnic minority group, it is important that there is diversity within those groups so that the participant group reflects the population being studied [8]. Simply including ethnic minorities in ongoing research, in the sense of recruiting a ‘quota’ of participants from a particular ethnic group for the population sample, is unlikely to add understanding. As those who are more accultured are more likely to participate, if there is a lack of appreciation of cultural differences and no consideration for the cultural and psychosocial influences on a participant, the research is unlikely to advance knowledge and may have little external validity [7]. Furthermore, factors such as an individual’s immigration status, family dynamics, and stigmatisation of mental illness may mean people are hesitant to take part in mental health research [9]; yet it is these populations within ethnic minority groups who subsequently are likely to be underrepresented. Intersectionality is a principle which proposes that we cannot see race, gender, and other social categories as separable. It challenges the idea of drawing conclusions in research based on just one categorisation, as generalising a finding to a whole population may be invalid due to the range of intracultural differences within a group [10]. Use of broad categories with no distinction between ethnicity and culture may mean research does not generate knowledge applicable to clinical practice; Cox and Simpson contended that ‘such an approach cannot enhance understanding of the complex interplay of social experience, health determinants, historical factors and power issues which inform high rates of morbidity and mortality’ [11].

### Language

The next theme emerging from the literature was language: studies were concerned with both literacy levels and with the language used– where it is not the first language of participants. Research materials which contain complex or scientific language may be barriers to recruitment and retention for those who are not easily able to comprehend this information [12], and subsequently mean there is a ‘gap’ in research that deepens the health inequalities affecting them. Particularly where assessment instruments, scales, or multiple-choice questions are being used, there can be an additional ‘cognitive burden’ for those who are not native speakers to understand what the question is asking; potentially leading to misinterpretation, even for those who would be considered fluent in the second language [13]. Where assessment tools have been translated, there can be problems of ‘translation equivalence’, where items may have a different meaning or connotation in the other language and the intended question or message is lost [14]. Literal translations or translation errors may render questionnaires incomprehensible and consequently result in the data being meaningless [15]. Olsen et al. [16] find that, despite using the recognised term for OCD in Spanish ‘Trastorno Obsesivo Compulsivo’, the community participating in the study identified the word ‘Trastorno’ as being negative and stigmatising, inferring severe mental illness; interestingly, in this study, pictures aided discussions about symptomatology. Even where translation equivalence is addressed, there is still the potential pitfall of conceptual equivalence, where the construct itself is not applicable cross-culturally. Communities may have their own idioms to describe their experiences, rather than conforming to the diagnostic criteria of psychiatric medicine [17]. Relying solely on the latter may lead to labelling distress as disease, mistaking culturally different behaviour for illness [18], and hence leading to incorrectly identifying higher rates of mental disorders.

### The influence of a research team on cultural sensitivity

Multiple papers suggest hiring research staff from the population being studied helps to ensure the research is designed in a culturally sensitive way [5, 19]. Participants may have been treated differently in everyday life because of their ethnicity, culture, class, gender, disabilities or sexuality [11], and may feel that researchers do not empathise with their life experiences [12]. Researchers should be aware of how their own personal characteristics may have an effect on participants and their perception of the study, and that there may be more reluctance to engage where a research team lacks shared characteristics or experiences with the participants [12]. Researchers may, even unconsciously, exhibit biases which affect participant responses [10] and it is prudent for individuals to examine their own attitudes prior to embarking on a study [20].

It may be practically impossible for research teams to recruit academics from each group being studied. However, there are alternative research methods that may allow for the meaningful participation of people from the ethnic background being studied to be part of the whole lifecycle of the research, such as adopting a framework of Community Based Participatory Research as discussed below. Derveeuw [21] cautions against the ‘tokenistic’ use of people of colour on research teams, and poignantly states ‘research benefits from diversity across the board, not just in skin colour’, indicating that academics tend to be from privileged groups and, therefore, including people working outside of academia will provide broader perspectives. Bishop et al. [22] suggest use of a ‘cultural consultant’ recommended by the community to provide advice about cultural appropriateness, who can also raise matters of concern and work to find solutions.

### Methodology

Many of the papers included in our review suggest that, when considering intersectionality and the heterogeneity of a population, the research methods chosen need to reflect these differences and a broad range of methodologies should be considered. Qualitative research can explore complexity and nuance in a way that is not generally captured through quantitative research methods; whilst this negatively affects generalisability, it has the potential to provide a richness of data [10, 23]. Whilst an evidence-based medicine approach favours quantitative methods, these can fail to capture subjective experiences and individual meaning, particularly in relation to mental health research [24]. Osafo [25] states that cultural psychological research requires ‘methods that allow people to talk about their experiences, more than simply sharing these experiences in numerical forms’. Mixed methods studies incorporate both qualitative and quantitative methods. When used together, these approaches can inform each other, creating a ‘feedback loop’’: where the data and results from qualitative research inform the quantitative research and vice versa, driving the development of the research [10]. Despite the endorsement of qualitative research as a culturally sensitive approach, it is still not always the ideal methodology. Wilson [26] cautions that qualitative research is ‘human centric’ and some indigenous communities see themselves as inseparable from nature, meaning that these methodologies may not be sufficient. Whilst exploring this is beyond the remit of this review, this is an interesting point to consider in highlighting the distinct cultural differences that can affect the process of research.

The literature describes several different frameworks to facilitate culturally competent research:

- Community Based Participatory Research (CBPR) is an approach which is ‘collaborative’ and involves creating an equitable platform with researchers for local community members, groups and organisations to co-create and progress the project [27]. This approach champions the ethos of ‘nothing about us without us’; it values different strengths, ensures the research is culturally appropriate and meets the needs of the local population by ensuring the local population are not only the researched, but also the researchers [28]. A CBPR approach may also improve studies’ internal and external validity and has been shown to improve outcomes for communities involved [27].
- The Campinha–Bacote model of cultural competence involves five constructs: cultural awareness, cultural knowledge, cultural desire, cultural skills and cultural encounters [29]. This model is applicable to every stage of research and starts with the researcher’s own self-awareness. This model is particularly applicable when translating an approach previously used in one ethnocultural population to another, different, population. Practically the researcher should be aware of their own beliefs and culture, have a receptive attitude and desire to gain knowledge about the population for the study, and apply strategies which capture culturally relevant data, for example capturing both explicit and implicit language expressions of the participants in interviews.
- The counselling lens approach is a ‘step-by-step’ approach to developing hypotheses, best used when previously accepted theory has cultural limitations, which ensures cultural validity going forward [30]. It entails examining the central constructs of the theory being tested, how the prior work that established that theory was conducted, and considering if more specific cultural constructs may be relevant.
- Fang tan is a tool to approach interviews with Chinese people, who may have a less individualistic and more interconnected understanding of the self, which is sometimes linked with less emotional openness. The principles consist of participant participation (dialogical conversation between the researcher and the participant), equality of status and insider relationship between the researcher and participant, as well as use of Chinese language.
- The cultural safety model aims to reduce power imbalances in healthcare and health research, considering social factors in health and the social position of healthcare professional researchers [11]. It recognises that culture is socially constructed, created by people, and is therefore changeable. Researchers need critically to reflect on their cultural positions, values and assumptions and address power relations between them and their participants, as well as thinking about how their research can challenge structural inequalities.
- The Relational Worldview model addresses a concept present within multiple cultures in which the four areas of mind, body, spirit, and social context are connected [31]. Using this model may mean researchers use a more diverse range of outcome measures in order to capture change more appropriately.

### Use of standardised instruments

Research often aims to measure mental wellbeing in a specific and comparable way through the use of standardised assessment tools and scales. For these tools to be used, it is important that they are culturally valid for the study population [5]. If an assessment tool has not been validated for the study group, then psychometric measures must be treated as a ‘tentative working hypothesis’ [15]. Instruments that have been established in one culture are not necessarily transferable to another culture and may not be culturally equivalent [14]; for example, interpersonal relationships are not frequently referenced in psychological tools, but are significant markers of wellbeing in some cultures [32]. The use of tools that are not culturally relevant in research may introduce measurement bias and reduce the validity of the study but can also lead to incorrect diagnosis and treatment for individuals [32–34]. It is important for researchers to consider whether tools can be transculturally translated with local meanings for concepts, or whether local cultural understandings of mental health are so unique that a standardised measure would fail to capture them accurately and a new scale needs to be developed [7, 18]. Assessments should ideally include standardised and non-standardised measures so that both generalisable and more nuanced, culturally specific data can be captured together and provide a richness to understanding [14].

### Ethical responsibilities of researchers

It is important to recognise that researchers bring their own biases to any research they conduct [23]. The previously mentioned cultural safety model suggests researchers must examine their own assumptions to explore which biases they bring to the research. This model goes further, calling on researchers to consider the roles of racism and colonisation in the ancestry of research and how they impact on the role of researcher and participant today [11]. There is a precedent of researchers acting as ‘safari-scholars’; swooping in to poke and prod, and then swiftly exiting [5], with indigenous communities being exploited and their knowledge marginalised through the process of research [26]. Some communities associate research with violence [26] and have halted their engagement with any researchers following negative experiences [35]. In terms of mental health research, Shore discussed the Barrow Alcohol Study in Alaska [19], which was billed a ‘cultural genocide’, as it shared a press release which shamed Inupiat communities for their levels of alcohol use and dependence [19]. The Tuskegee syphilis study is infamous for its abuses of African American communities and has led to ‘deep mistrust of academic and research institutions and investigators’ [36]. Despite this historical backdrop, ethnic minority groups can be labelled as difficult to recruit or ‘hard to reach’, without recognising the generational trauma that exists in the racist history of mental health research. It is important to consider who the research is for [35] and ‘whether research is dominated by certain cultural agendas, views or positions’ [11].

When the study is relevant, researchers are sensitive, transparent, and effective in their communication, then ethnic minorities are indeed willing to participate [36]. Participants value research communities [35] where they have the ability to control the direction of the research [5]. Community involvement should start from the planning stages of research and continue throughout for it to be meaningful [19]. All team members should seek knowledge of the culture of the community in which the study will take place [37].

There is a duty to gain informed consent, in a culturally responsible way, considering the potential for inadvertent coercion [37], and to maintain confidentiality and anonymity of the community if examining a small, easily identifiable population [19]. It is prudent to be aware of the effect that the research has on the participants, as research is a process which can also have an effect on the study population and cause a change in and of itself [38]. When the study ends, the results should be shared with the participants [19], and debriefing participants can reduce any negative impact of the study [35].

## Discussion

This scoping review aimed to understand how mental health research can be conducted in a way that is meaningfully (as opposed to tokenistically) culturally sensitive. This is important so that research findings reflect the human experience, not just an insular understanding of it: and to reduce disparities in mental health and its treatment. This review has highlighted the fact that that truly culturally sensitive research requires commitment from its inception: as Trimble [5] states “without a conscious intent and desire, the achievement and realisation of cultural competence is not likely to occur”. Culturally sensitive research does not imply ticking a set of boxes which can be applied to make a study more palatable, or a paragraph paying lip service to cultural differences. Rather, it is an approach that involves critical analysis, self-reflection, collaboration and a willingness to change applied at every stage of conception and planning. This may require researchers to be stepping out of their ‘comfort zones’, being adaptable to new ways of working, and being responsive to concerns during the process. This responsibility lies not only with individual research teams, but also with institutions. Ethics committees should include those with an understanding of minority groups [38, 39] and, where there are research guidelines, there is a need for these to be applicable cross-culturally [40]. Whilst specifically approaching indigenous communities may not to be relevant to all research, all research is cultural research [13], and all researchers have a responsibility to be culturally sensitive.

In mental health, we frequently rely on subjective descriptions of symptomology in the clinical setting. Therefore, it is essential to consider the cultural factors which have influenced the person’s background and current presentation. It is evident from the literature that those from different cultural backgrounds may perceive the concept of mental illness in a way that does not align with a ‘Western’ medical model, and they may have different ways of expressing and coping with distress [18]. There are many alternative models and adjustments suggested that researchers can use to increase our understanding.

### Strengths and limitations

We searched many different databases, including grey literature, meaning that the searches were comprehensive, adding strength to the findings.

We realised that the review itself will be influenced by the biases of the researchers, most of whom were young, female, white professionals. Although we did utilise grey literature to expand the types of studies we included, we relied on traditional academic methods and mainstream databases to conduct the review. It is possible that paper records, books, social media or other forms of data of which we are unaware would have provided us with different insights, that were not considered because of the search methods we used. We were less likely to find papers that use these kinds of methodologies because they are considered less ‘high quality’ and may not have been in the types of databases we were searching. Even word of mouth or storytelling are key methods of dialogue between some communities [26]. It is surprising that all the studies were in English, despite no language restrictions. This may indicate a publication bias favouring English in academic literature or suggest that terms related to “cultural competence” are more commonly found in English-language sources.

## Conclusions

This work has highlighted the challenge of capturing a diverse range of experiences and perspectives in order to ensure that research in health represents the population for whom it is intended. There will always be limitations when working within established medical research frameworks. However, there are straightforward ways in which this can be culturally adapted, starting with researchers candidly examining their own ideas about culture so that they can begin more easily to create a culturally appropriate research environment for participants. If we are to steer academia towards more equitable research and research outputs, that are meaningful and likely to change the disparities we see along ethnic lines, we need to challenge it's history of association with colonialism. This includes understanding that experiences of others do not necessarily fit within our rubric of mental illness or mental ‘disorder’ and that these can inadvertently perpetuate a narrative of imperialism. Given the abuses of ethnic minorities in research (compounded by the history of eugenics in psychiatry), individuals have cause to be wary about recurring racism. The onus is on clinicians and researchers to acknowledge past abuses, and the social and cultural determinants of health, rather than dissociating themselves from it. The disparities in prevalence of illness, in timely access to care and appropriate treatment that persist for some communities, suggest an urgent need to further our understanding through culturally informed research so that we can create mental health services where ethnic minority groups can get the care that is best for them at the point of need.

## Electronic supplementary material

Below is the link to the electronic supplementary material.

Supplementary Material 1

## Data Availability

The datasets used and/or analysed during the current study are available from the corresponding author on reasonable request. The searches used are available in a data repository available at: 10.48420/26357215.v1.
