# Supplementary material for: Culturally sensitive mental health research: a scoping review
Source: BMC Psychiatry. 2025 Mar 3;25:190. doi: 10.1186/s12888-025-06575-z (PMC11874804; doi:10.1186/s12888-025-06575-z)
Supplement: Supplementary file 1 — Supplementary Material 1 [file 12888_2025_6575_MOESM1_ESM.docx]

Example Search Strategy

1 (CULTUR* COMPETEN* or CULTUR* SENSITIV* or CULTUR* AWARE* or CULTUR* APPROPRIATE* or CULTUR* EDUCAT* or CULTUR* CONGRUEN* or CULTUR* BIAS* or CULTUR* RESPONS* or CULTUR* UNDERSTAND*).ab

2 (CULTUR* COMPETEN* or CULTUR* SENSITIV* or CULTUR* AWARE* or CULTUR* APPROPRIATE* or CULTUR* EDUCAT* or CULTUR* CONGRUEN* or CULTUR* BIAS* or CULTUR* RESPONS* or CULTUR* UNDERSTAND*).ti

3 exp cultural sensitivity/

4 (PSYCH* RESEARCH or PSYCH* HEALTH RESEARCH or MENTAL HEALTH RESEARCH or MENTAL DIS* RESEARCH or EMOTIONAL HEALTH RESEARCH or PSYCH* STUD* or MENTAL HEALTH STUD* or MENTAL DIS* STUD* or EMOTIONAL HEALTH STUD* or PSYCH EXPERIMENT* or MENTAL HEALTH EXPERIMENT* or MENTAL DIS* EXPERIMENT* or EMOTIONAL HEALTH EXPERIMENT* or RESEARCH ABOUT MENTAL DIS* or RESEARCH ABOUT MENTAL HEALTH or RESEARCH ABOUT PSYCH* or RESEARCH ABOUT EMOTIONAL HEALTH or RESEARCH ON MENTAL DIS* or RESEARCH ON MENTAL HEALTH or RESEARCH ON PSYCH* or RESEARCH ON EMOTIONAL HEALTH or MENTAL DISORD* RESEARCH or EMOTIONAL HEALTH STUD* or EMOTIONAL HEALTH RESEARCH).ab

5 (PSYCH* RESEARCH or PSYCH* HEALTH RESEARCH or MENTAL HEALTH RESEARCH or MENTAL DIS* RESEARCH or EMOTIONAL HEALTH RESEARCH or PSYCH* STUD* or MENTAL HEALTH STUD* or MENTAL DIS* STUD* or EMOTIONAL HEALTH STUD* or PSYCH EXPERIMENT* or MENTAL HEALTH EXPERIMENT* or MENTAL DIS* EXPERIMENT* or EMOTIONAL HEALTH EXPERIMENT* or RESEARCH ABOUT MENTAL DIS* or RESEARCH ABOUT MENTAL HEALTH or RESEARCH ABOUT PSYCH* or RESEARCH ABOUT EMOTIONAL HEALTH or RESEARCH ON MENTAL DIS* or RESEARCH ON MENTAL HEALTH or RESEARCH ON PSYCH* or RESEARCH ON EMOTIONAL HEALTH or MENTAL DISORD* RESEARCH or EMOTIONAL HEALTH STUD* or EMOTIONAL HEALTH RESEARCH).ti

6 exp mental health research/

7 4 or 5 or 6

8 1 or 2 or 3

9 7 and 8
